# Immobilization of Zinc Phthalocyanines in Silicate Matrices and Investigation of Their Photobactericidal Effect on E.coli

**DOI:** 10.1100/tsw.2006.75

**Published:** 2006-03-26

**Authors:** Spas Artarsky, Stanislava Dimitrova, Raymond Bonnett, Milka Krysteva

**Affiliations:** Department of Biotechnology, University of Chemical Technology and Metallurgy, Sofia, Bulgaria; School of Biological and Chemical Sciences, Queen Mary, University of London, UK

**Keywords:** phthalocyanine, photobactericidal action, immobilization

## Abstract

The aim of the present investigation was to immobilize zinc phthalocyanines in a silicate matrix and to test the photobactericidal properties of the matrices so prepared toward Esherichia coli in model aqueous media. For the purpose, tetra tertiary butyl zinc phthalocyanine (TBZnPc) and zinc phthalocyanine tetrasulfonic acid (ZnPcTS) were used. The abilities of these two photosensitizers to generate singlet oxygen in solution were compared by following the rate of photobleaching of 1,3-diphenylisobenzofuran (DPBF) at 430 nm in dimethylformamide (DMF).The results of this study show clearly that, under the conditions used here, the TBZnPc is the more effective generator of singlet oxygen; with it the DPBF was virtually completely photobleached in 4 min, while with the ZnPcTS under the same conditions, it took 12 min to reach this point. Glass conjugates with the two phthalocyanines were obtained by the sol-gel technique and were characterized by a well-defined color due to the phthalocyanine incorporated in the silicate matrix. Glasses with an intense, but inhomogeneous, green color were obtained when the tetrasulfonic derivative of the zinc phthalocyanine was used, while blue glasses of evenly distributed coloration were formed from the tetra tertiary butyl derivative.The ZnPcTS conjugate demonstrates more effective singlet oxygen evolution than is the case with the TBZnPc conjugate. These results are the opposite of those obtained for the free phthalocyanines in solution. The structural formulae of the compounds show that TBZnPc has a more pronounced hydrophobic character than the sulfonic derivative. In our view, the relative reactivities of the conjugates can be explained by the tetrasulfonic derivative being situated mainly in the surface parts of the glass matrix where the hydrophilic character is prevailing, while the tertiary butyl derivative is mainly present in the internal parts of the matrix as a result of which it is less accessible and therefore less activeThe results obtained on the effect of zinc phthalocyanine conjugates on E. coli show a trend similar to that observed with singlet oxygen evolution shown. Thus, for the ZnPcTS conjugate, the log kill is 1.32 and for the TBZnPc conjugate, it is 0.98, in each case after 120 min. The results obtained show that phthalocyanines can be immobilized successfully in a silicate matrix and used for photodisinfection of microbially polluted waters. The silicate matrix has some advantages in comparison with other organic matrices. It is insoluble in water, resistant towards microorganisms, easy to fabricate, and might be developed successfully for the photodisinfection of water, e.g., in swimming pools and in other open water reservoirs.

## INTRODUCTION

Phthalocyanines are photosensitizers that are capable of absorbing light energy to give excited states that are, in turn, able to generate reactive oxygen species, and in particular singlet oxygen, by energy transfer to oxygen molecules. These reactive oxygen species are toxic to living cells (photodynamic action), a process that includes photodisinfection[1]. Various porphyrins and xanthene dyes (e.g., eosin[2], rose Bengal[3,4]) also behave as photosensitizers in this way.

Such photosensitizers have found application, or have potential, in the photodynamic therapy of tumors[5]; the inactivation of viruses, yeasts, and bacteria; the production of surgical materials; and the treatment of psoriasis and arthritis[6,7].

The present investigation was aimed at immobilizing zinc phthalocyanines in a silicate matrix and testing the photobactericidal properties of the matrices so prepared toward *Esherichia coli* in model aqueous media. Related photosensitizer-impregnated siloxane polymers have been used previously; for example, in the study of singlet oxygen reactions with olefins[8] and in model wastewater clean-up studies using 2-chlorophenol as the target[9].

## RESULTS AND DISCUSSION

### Investigation of the Photosensitizing Properties of the Phthalocyanines Used

The chemical structures, names, and acronyms of the phthalocyanines investigated here are shown in Fig 1.

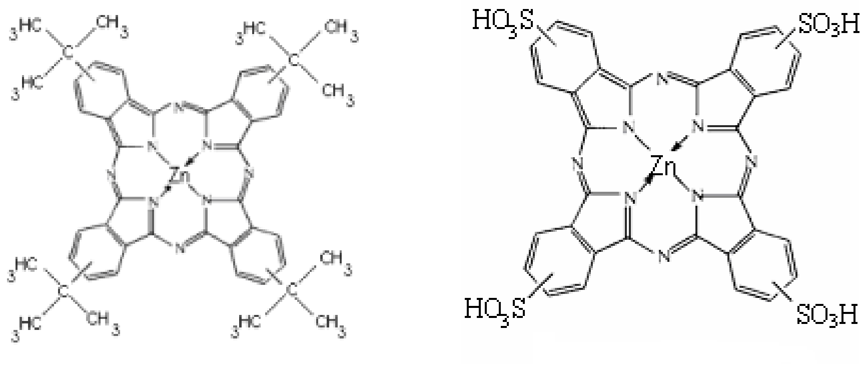

**A** **B**

**FIGURE 1**. Chemical structures of the photosensitizers: (A) tetra tertiary butyl zinc phthalocyanine (TBZnPc), (B) zinc phthalocyanine tetrasulfonic acid (ZnPcTS).

The abilities of these two photosensitizers to generate singlet oxygen in solution were compared by following the rate of photobleaching of 1,3-diphenylisobenzofuran (DPBF) at 430 nm in dimethylformamide (DMF).

The results of this study are presented in Fig. 2, which shows clearly that under the conditions used here, the TBZnPc is the more effective generator of singlet oxygen; with it the DPBF is virtually completely photobleached in 4 min, while with the ZnPcTS under the same conditions, it takes 12 min to reach this point.

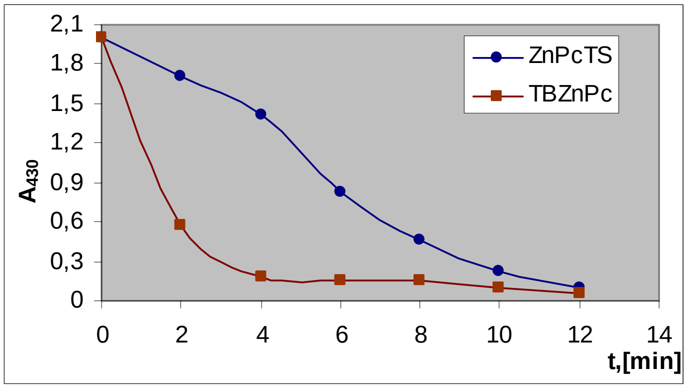

**FIGURE 2**. Time course of photobleaching of DPBF by TBZnPc and ZnPcTS.

It may be concluded that with these phthalocyanines in solution, the TBZnPc derivative shows a much more pronounced photosensitizing effect as compared to the tetrasulfonic derivative.

## Characterization of the Conjugates

Glass conjugates with the two phthalocyanines shown in Fig. 1 were obtained by the sol-gel technique (described in the experimental section). These conjugates were characterized by a well-defined color that is due to the phthalocyanine incorporated in the silicate matrix. Glasses with an intense, but inhomogeneous, green color were obtained when the tetrasulfonic derivative of the zinc phthalocyanine was used, while blue glasses of evenly distributed coloration were formed from the tetra tertiary butyl derivative. Photographs of fragments of these two kinds of conjugates are shown in Fig. 3; the largest fragments are about 1 cm in length.

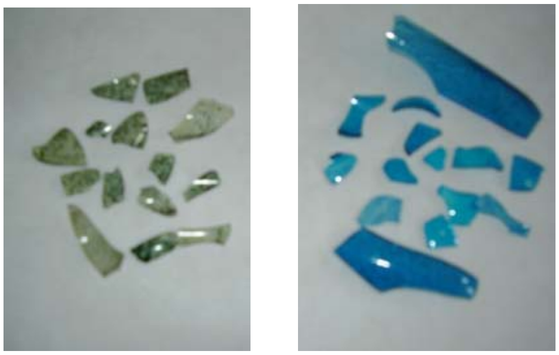

A B

**FIGURE 3**. Photograph of the conjugates obtained: (A) conjugate of ZnPcTS; (B) conjugate of TBZnPc.

## Investigation of the Photosensitizing Properties of the Conjugates

The photosensitizing properties of the two colored glasses were initially examined by investigating the photobleaching of DPBF. For that purpose, 0.5 g of each conjugate was suspended in DMF. The control was a colorless glass prepared by the same sol-gel procedure, but without added photosensitizer. The results obtained are given in Fig. 4.

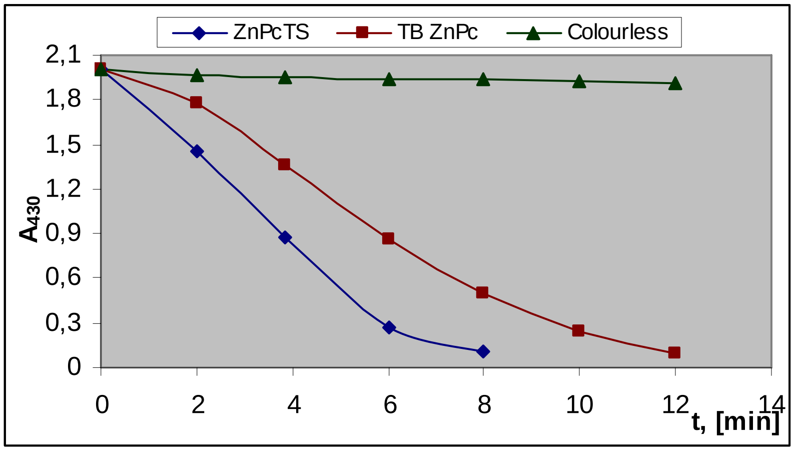

**FIGURE 4**. Kinetic studies of the photobleaching of DPFB by TBZnPc and ZnPcTS conjugates suspended in DMF.

As is evident from the figure, the ZnPcTS conjugate demonstrates more effective singlet oxygen evolution than is the case with the TBZnPc conjugate. These results are the opposite of those obtained for the free phthalocyanines in solution (Fig. 2). The structural formulae of the compounds (Fig. 1) show that TBZnPc has a more pronounced hydrophobic character than the sulfonic derivative. In our view, the relative reactivities of the conjugates can be explained by the tetrasulfonic derivative being situated mainly in the surface parts of the glass matrix where the hydrophilic character is prevailing, while the tertiary butyl derivative is mainly present in the internal parts of the matrix as a result of which it is less accessible and therefore less active. These hypothetical structures are shown in Fig. 5.

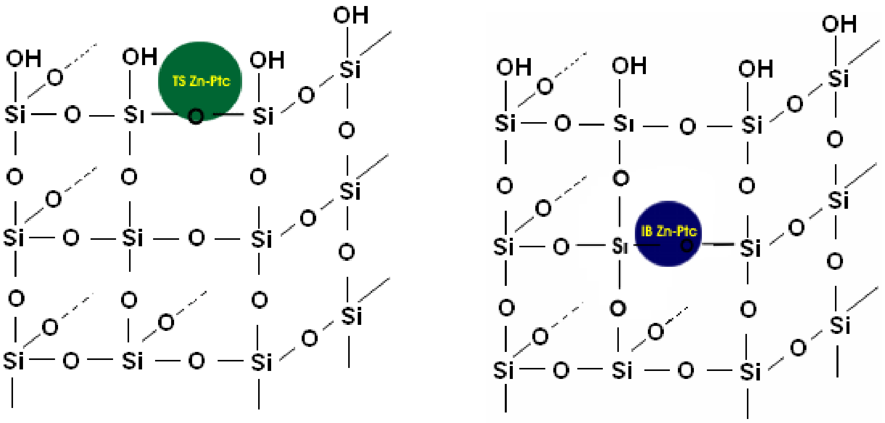

**A** **B**

**FIGURE 5**. Hypothetical structures of silicate conjugates with: (A) ZnPcTS; (B) TBZnPc (diagrammatic only).

## Investigation of the Photobactericidal Properties of the Conjugates

The experimental setup is shown in Fig. 6.

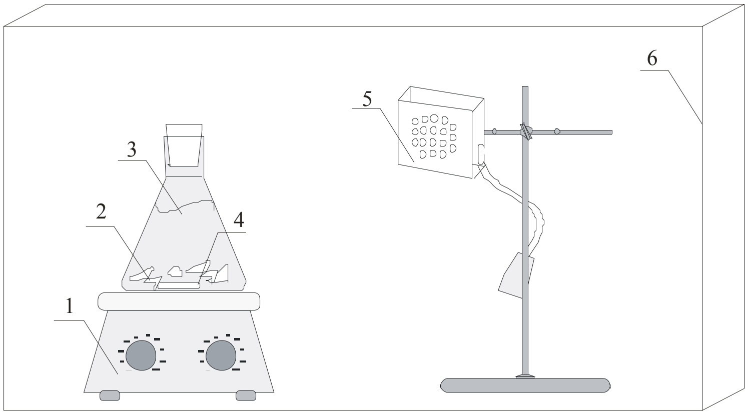

**FIGURE 6.** Scheme of the experimental apparatus: 1, stirrer; 2, fragments of glass conjugates; 3, reactor; 4, magnetic stirrer; 5, bank of light-emitting diodes; 6, dark box.

A microbial culture of *E. coli* with a $10^6$ cfu/ml concentration (cfu = colony forming units) was used for the experiment. The experiment was carried out with 1 g of each conjugate material, and colorless glasses were used as controls. The contact between the conjugates and the microbial suspension was achieved in reactor (3) under irradiation from the light-emitting diode lamp (5) at a distance of 25 cm. The whole device was placed in a dark box. The phthalocyanines in the silicate matrix (2) generated singlet oxygen exercising a photobactericidal effect on the microorganisms. Surviving microorganisms in the solution were measured (as cfu) by taking serially diluted samples (standard volume 0.1 ml) at definite time intervals and plating them on solid agar (an "Endo" solid nutrient medium). After 24 h of cultivation, the surviving microorganisms were counted. The estimations were done in triplicate and averaged and the photobactericidal effect was plotted on a logarithmic scale. The error is estimated to be ±4%. The results for the two conjugates are shown in Figs. 7 and 8.

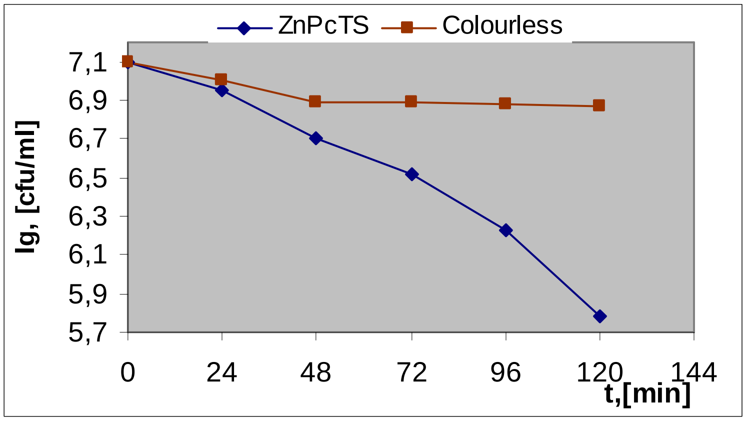

**FIGURE 7.** Kinetics of photodisinfection of a microbial solution with the ZnPcTS conjugate.

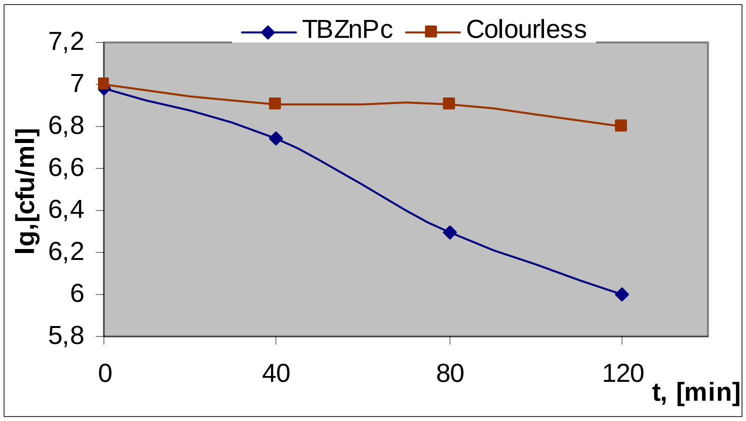

**FIGURE 8.** Kinetics of photodisinfection of a microbial solution using the TBZnPc conjugate.

*E. coli* was selected as a test microorganism because it is known to be a widespread microbial pollutant of surface waters, and its levels are used as a pollution indicator[10,11].

Although the photodynamic action is apparently much slower in the microbial experiment than in the DPBF experiment (presumably because the activity now has two barriers to cross), the results obtained on the effect of zinc phthalocyanine conjugates on *E. coli* show a trend similar to that observed with singlet oxygen evolution shown in Fig. 4. Thus, for the ZnPcTS conjugate, the log kill is 1.32 and for the TBZnPc conjugate, it is 0.98, in each case after 120 min.

The photobleaching of DPBF observed with both phthalocyanines (TBZnPc, ZnPcTS) accords with a singlet oxygen mechanism, as first clarified by Foote[12 ] in which electronic excitation energy is transferred from the excited triplet of the sensitizer to triplet dioxygen, to give the sensitizer in its ground state and singlet oxygen thus:

$$\text{Sens}\ (S_0) \xrightarrow{h\nu} \text{Sens}\ (T_1)$$

$$\text{Sens}\ (T_1) + {}^3O_2 \longrightarrow \text{Sens}\ (S_0) + {}^1O_2$$

$$A + {}^1O_2 \longrightarrow AO_2$$

where “A” in our case is either DPBF or *E. coli* cells.

On the basis of this mechanism, we suppose that the accessibility of ZnPcTS conjugate to both light energy and its surroundings is much better expressed than that of TBZnPc because of differences in hydrophilicity. So ZnPcTS conjugate under light illumination can interact better with triplet dioxygen than can TBZnPc.

It is evident that soluble photosensitizers will interact under illumination with triplet dioxygen depending only on their structural features. For immobilized photosensitizers, another factor appears and it is their accessibility to triplet dioxygen. In our opinion, this is the reason for the difference of the behavior of photodynamic action of phthalocyanine conjugates studied.

The stability of the colored glasses obtained with respect to the effect of daylight was also subjected to investigation. For that purpose, equal amounts of the colored glasses were exposed to daylight for 72 h, the samples then being evaluated by the DPBF technique. It was found that exposure to daylight for 72 h did not affect the ability of the conjugate glasses to generate singlet oxygen.

## EXPERIMENTAL METHODS AND PROCEDURES

- Tetraethylorthosilicate (TEOS) was obtained from Merck (Germany), and 1,3-diphenylisobenzofuran (DPBF) from Sigma-Aldrich. All the remaining reagents were AR.
- ZnPcTS[13] and TBZnPc[14] were synthesized by literature methods.
- *E. coli* 1089 were bought from the National Bank for Microorganisms and Cell Cultures, Sofia.
- The light source was a Bonnett-Pell lamp, being a bank of 49 light-emitting diode sources [Kingbright Superbright red (AlGaAs), code L53SRCE], maximum emission at 660 nm, mounted within a circle of 6-cm diameter and operated at 20 mA, giving a fluence rate at 25 cm = 0.60 mW $cm^{-2}$.

### Immobilization of Phthalocyanines in the Silicate Matrix by the Sol-Gel Procedure

A. **Immobilization of ZnPcTS** — The phthalocyanine (2 mg) was dissolved in 30-ml ethanol with stirring using a magnetic stirrer. The phthalocyanine solution and one to two drops of concentrated hydrochloric acid were added to 10-ml TEOS in 30-ml ethanol. The mixture obtained was stirred and transferred to a Petri dish. After 3 days, evaporation fragments of greenish glass (Fig. 3) were obtained.

B. **Immobilization of TBZnPc** — The phthalocyanine (2 mg) was dissolved in 10-ml DMF using a magnetic stirrer for 30 min. TEOS (10 ml) was dissolved in 30-ml ethanol and the phthalocyanine solution along with one to two drops of concentrated hydrochloric acid were added to the solution. The mixture was stirred to achieve homogenization, then transferred to a Petri dish, and allowed to stand at room temperature for 3 days until the formation of fragments of transparent bright blue glass (Fig. 3).

### Investigation of the Photoactivity of the Conjugates Using DPBF[15]

The investigation was performed with 0.5-g conjugate material, which was placed into 50-ml 0.1-m*M* DPBF solution of DMF. The latter was irradiated with a bank of light-emitting diodes, 660 nm, at a distance of 25 cm in an otherwise dark chamber during continuous stirring with a magnetic stirrer. As a result of the irradiation, the phthalocyanine conjugates generated singlet oxygen, which oxidized DPBF. Samples were withdrawn at definite time intervals and optical densities were measured at 430 nm using a Perkin Elmer Lambda 2 IR-VIS (Fig. 4).

An analogous procedure was used to study the photoactivities of the free phthalocyanines in solution (Fig. 2).

### Cultivation of *Esherichia coli* Microorganisms

A. **In liquid nutrient medium** — *E. coli* microorganisms at the end of the logarithmic phase of growth were introduced into a sterilized liquid nutrient medium MPB 100 ml, placed in a sterile box. The microbial sample was cultivated for 16–18 h. Cultivation proceeded with shaking at 37°C.

B. **In solid nutrient medium** — Sterilized Petri dishes, 10–12 cm in diameter, containing sterilized “Endo” solid selective nutrient medium, were allowed to stand in a thermostat for 24 h, then, in the presence of sterilized medium, a 100-μl sample was transferred onto the center of the Petri dish, and then spread over the whole surface. The procedure was carried out in a sterile box. The microbial specimen obtained was cultivated for 24 h in a thermostat at a temperature of 37°C, and the colonies were then counted.

## CONCLUSION

The results obtained show that phthalocyanines can be immobilized successfully in a silicate matrix and used for photodisinfection of microbially polluted waters. The silicate matrix has some advantages in comparison with other organic matrices. It is insoluble in water, resistant towards microorganisms, easy to fabricate, and relatively inexpensive to produce. The two phthalocyanine conjugates (Fig. 3) investigated show similar activities; the ZnPcTS conjugate having (Fig. 4) a slightly more pronounced photobleaching effect against DPBF compared with the TBZnPc conjugate. This may be attributed to the difference in the hydrophilic properties of the sensitizing molecules. In photomicrobicidal activity against *E. coli*, the conjugates follow the same order. With suitable development to increase the rate of the effect and the log kill, it is proposed that sol-gel glasses containing phthalocyanine or other photosensitizers might be successfully developed for the photodisinfection of water (Figs. 7 and 8), e.g., in swimming pools and in other open water reservoirs.

## BIOSKETCHES

**Spas Artarsky** is a PhD student in the Department of Biotechnology, University of Chemical Technology and Metallurgy, Sofia, Bulgaria.

**Stanislava Dimitrova** is a diploma student in the Department of Biotechnology, University of Chemical Technology and Metallurgy, Sofia, Bulgaria.

**Raymond Bonnett, PhD (Cantab.), DSc (London),** is Emeritus Professor of Organic Chemistry at the School of Biological and Chemical Sciences, Queen Mary, University of London, Mile End Road, London E1 4NS, U.K. E-mail R.Bonnett@qmul.ac.uk. His interests include the chemistry and biochemistry of porphyrins and related compounds (haem, chlorophyll, bile pigments, and vitamin $B_{12}$): in recent years he has been engaged in work on the applications of photodynamic therapy and is codiscoverer of the photodrug 'Foscan'®, a chlorin derivative that is approved in Europe for the treatment of head and neck cancer.

**Milka Krysteva, PhD, DSc,** is a Professor in the Department of Biotechnology, University of Chemical Technology and Metallurgy, 8 Kl.Ohridski str. Sofia 1756, Bulgaria; Tel/Fax: 359 2 868 34 60; E-mail: krysteva@uctm.edu. Lecture courses include applied enzymology and chemistry of biologically active compounds. Research interests are enzymes: structure and function; immobilization of enzymes; preparation of membranes with immobilized enzymes for biosensors; application of immobilized enzymes for detection of different compounds; immobilization of cells and their application for production of different steroids, biogas, and hydrogen; and immobilization of photosensitizers and application for water sterilization.
